# Preventing and lessening exacerbations of asthma in school-age children associated with a new term (PLEASANT): study protocol for a cluster randomised control trial

**DOI:** 10.1186/1745-6215-14-297

**Published:** 2013-09-16

**Authors:** Michelle J Horspool, Steven A Julious, Jonathan Boote, Mike J Bradburn, Cindy L Cooper, Sarah Davis, Heather Elphick, Paul Norman, W Henry Smithson, Tjeerd vanStaa

**Affiliations:** 1 Clinical Trials Research Unit, University of Sheffield, School of Health and Related Research, 30 Regent Street, Sheffield S1 4DA, UK; 2 Medical Statistics Group, University of Sheffield, School of Health and Related Research, 30 Regent Street, Sheffield S1 4DA, UK; 3 Design, Trials and Statistics, University of Sheffield, School of Health and Related Research, 30 Regent Street, Sheffield S1 4DA, UK; 4 Health Economics and Decision Science, University of Sheffield, School of Health and Related Research, 30 Regent Street, Sheffield S1 4DA, UK; 5 Department of Paediatric Respiratory Medicine, Sheffield Children’s Hospital, Western Bank, Sheffield S10 2TH, UK; 6 Department of Psychology, University of Sheffield, Western Bank, Sheffield S10 2TP, UK; 7 Academic Unit of Primary Medical Care, University of Sheffield, Samuel Fox House, Northern General Hospital, Herries Road, Sheffield S5 7AU, UK; 8 Clinical Practice Research Datalink, Medicines and Healthcare Products Regulatory Agency, 5th Floor, 151 Buckingham Palace Road, Victoria, London SW1W 9SZ, UK

**Keywords:** Asthma, Childhood asthma, Asthma exacerbation, Cluster randomised controlled trial, Primary care, Public health intervention, Unscheduled medical appointments

## Abstract

**Background:**

Within the UK, during September, there is a pronounced increase in the number of unscheduled medical contacts by school-aged children (4–16 years) with asthma. It is thought that that this might be caused by the return back to school after the summer holidays, suddenly mixing with other children again and picking up viruses which could affect their asthma. There is also a drop in the number of prescriptions administered in August. It is possible therefore that children might not be taking their medication as they should during the summer contributing to them becoming ill when they return to school.

It is hoped that a simple intervention from the GP to parents of children with asthma at the start of the summer holiday period, highlighting the importance of maintaining asthma medication can help prevent increased asthma exacerbation, and unscheduled NHS appointments, following return to school in September.

**Methods/design:**

PLEASANT is a cluster randomised trial. A total of 140 General Practices (GPs) will be recruited into the trial; 70 GPs randomised to the intervention and 70 control practices of “usual care”. An average practice is expected to have approximately 100 children (aged 4–16 with a diagnosis of asthma) hence observational data will be collected on around 14000 children over a 24-month period. The Clinical Practice Research Datalink will collect all data required for the study which includes diagnostic, prescription and referral data.

**Discussion:**

The trial will assess whether the intervention can reduce exacerbation of asthma and unscheduled medical contacts in school-aged children associated with the return to school after the summer holidays. It has the potential to benefit the health and quality of life of children with asthma while also improving the effectiveness of NHS services by reducing NHS use in one of the busiest months of the year.

An exploratory health economic analysis will gauge any cost saving associated with the intervention and subsequent impacts on quality of life. If results for the intervention are positive it is hoped that this could be adopted as part of routine care management of childhood asthma in general practice.

**Trial registration:**

Current controlled trials: ISRCTN03000938 (assigned 19/10/12) http://www.controlled-trials.com/ISRCTN03000938/.

UKCRN ID: 13572

## Background

Asthma exacerbations and deaths are known to be seasonal [1]. A number of reports have shown peaks in asthma exacerbation in school aged children with asthma associated with the return to school following the summer vacation [2-10]. These studies mainly report hospital admissions, although one study has reported peaks both in hospital admissions and all medical contacts [10].

Children returning to school are exposed to a variety of novel respiratory challenges including allergens and viruses, at a time of changing climactic conditions. It has previously been shown that viral infection and allergen exposure in allergen sensitised asthmatics are associated with increased hospital admissions for acute asthma [11]. The same study demonstrated the protective effect of inhaled corticosteroids on acute asthma exacerbations in a paediatric asthma population [11].

**Figure 1:**
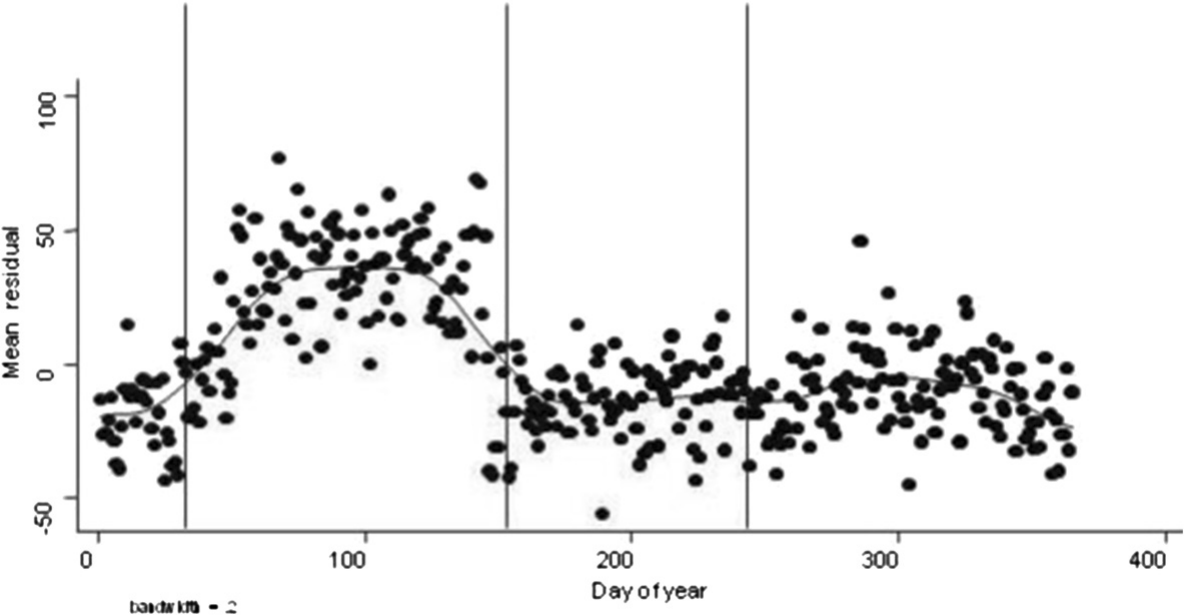
**Mean residuals for excess medical contacts for children with asthma for over controls in England.** The vertical lines represent, from left to right, the 1st September, 1st January and 1st April with LOESS smoothing curve that enables trends to be seen [13].

In previous research by the study team a random sample of around 75,000 school age (5–16 years) children were observed, using a data set from selected general practices within the General Practice Research Database (now the Clinical Practice Research Datalink [12]), who had a documented medical diagnosis of asthma. Age (within 2 years) and sex matched controls from the same practice were also taken [13]. The investigation confirmed the increase in unscheduled medical contacts in children with asthma throughout the year with an approximate doubling in medical contacts compared to non-asthmatic children. Regression analysis showed that children with asthma were approximately twice as likely as controls to have unscheduled medical contact and were more likely to see their doctor around the return to school. If asthmatic children were at a constant increased risk of medical contacts throughout the year Figure 1 would show a random scatter of the residuals in England. However, around the return to school there was a pronounced positive increase in the value of residuals (a similar pattern was observed for Scotland). This indicates that at this time there was a greater than expected increase in the number of contacts by children with asthma compared to controls.

It could therefore be argued that July and August are a good time to have asthma; the pollen season is almost over, school age children are not at school and so have less opportunity to pick up any viral infections that are going through the population.

**Figure 2:**
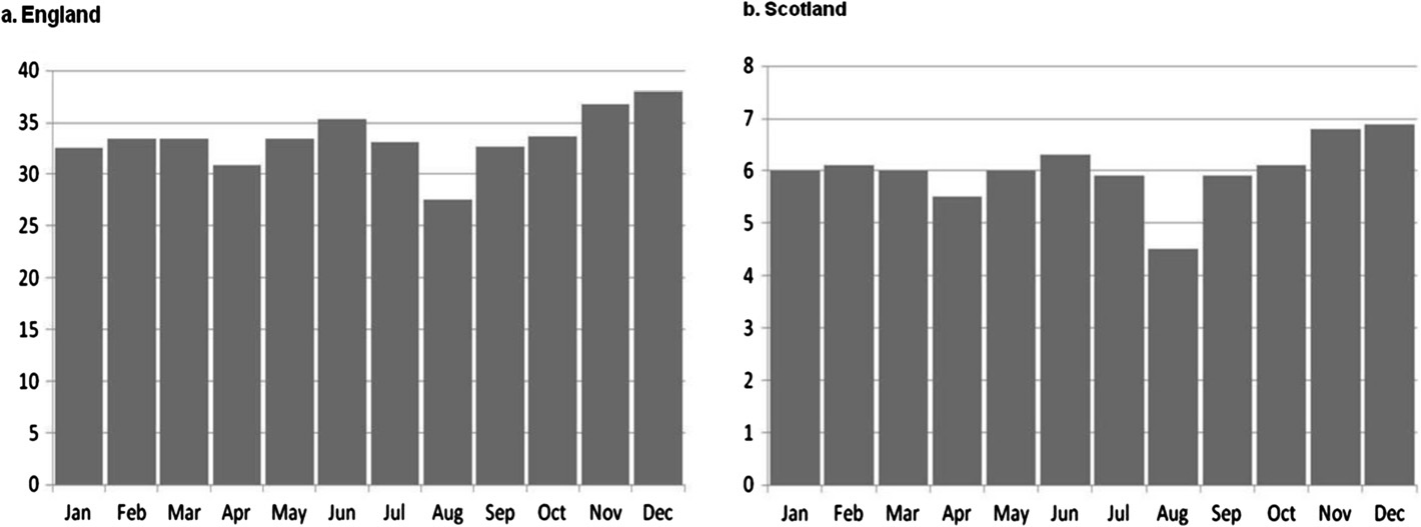
Average daily prescriptions by month for England and Scotland.

In the same study we also observed a drop in prescriptions for inhaled steroids in August immediately preceding the return back to school with 25% fewer prescriptions in August compared to July and September [13] (see Figure 2). This drop in prescriptions precedes the viral challenge of a return back to school. We further showed that patients who received a prescription for inhaled corticosteroid had 0.14 fewer contacts per patient (95% CI 0.12 to 0.16, P < 0.001, England; 95% CI 0.10 to 0.18, P < 0.001, Scotland) than those who did not receive an August prescription.

To interpret the figure of 0.14: hypothetically imagine a cohort of 200 children with asthma on inhaled corticosteroids, where 100 receive an August prescription and 100 do not. If the 100 patients with a prescription make a total of 50 unscheduled medical visits (0.5 mean visits/patient) then 64 unscheduled medical contacts would be made by those not receiving a prescription (0.64 mean visits/patient; difference 0.14). Hence, per 100 children with asthma on inhaled corticosteroid not receiving a prescription in August there is an excess of 14 unscheduled medical contacts. Unplanned medical contacts cost the NHS: £36 for a contact in surgery; £121 for a GP home visit [14]; £59 to £142 for an emergency department contact if not admitted and £74 to £249 if admitted; £385 for a non-elective short stay for asthma without complications [15].

It is therefore likely that children who stop taking or reduce their inhaled corticosteroids over the summer months or run low of other medications, and fail to restart them before the return to school, are more at risk of acute asthma exacerbation.

The PLEASANT trial will be observing the effect of a brief postal intervention sent to school aged children (4–16) with asthma. A letter, will be sent by their General Practitioner (GP) at the start of the school summer holidays, encouraging continuation with their prescribed asthma medication before the start of the new school year. We will assess the impact this has on subsequent unscheduled NHS contacts and reduction in exacerbation of asthma.

## Methods/Design

### Study design

**Figure 3:**
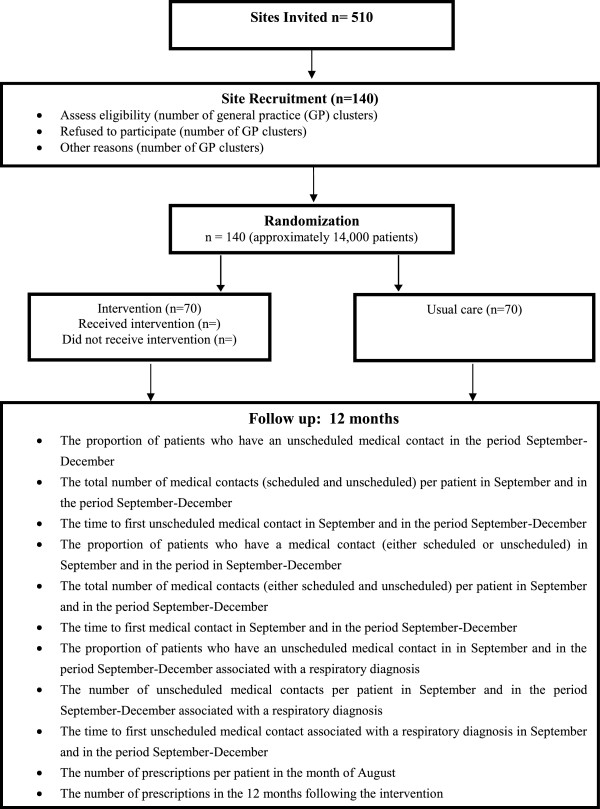
Trial summary.

PLEASANT is a cluster randomised trial. A total of 140 General Practices (GPs) will be recruited into the trial; 70 GPs randomised to the intervention and 70 control practices of “usual care” (Figure 3). An average practice is expected to have approximately 100 children eligible hence data will be collected on over 14000 children over a 12 month period. This is a non-commercial, portfolio study adopted by the Primary Care Research Network.

### Primary research objective

The aim of the trial is to assess if a brief postal intervention reduces the number of unscheduled medical contacts after the return to school. The primary objective of the study is to assess whether the intervention reduces the September peak in total medical contacts and subsequent exacerbations in asthma.

### Primary outcome measure

1. The proportion of patients aged between 5–16 who have an unscheduled medical contact in September

### Secondary outcome measures

1. The proportion of patients who have an unscheduled medical contact in the period September-December

2. The total number of medical contacts (scheduled and unscheduled) per patient in September and in the period September-December

3. The time to first unscheduled medical contact in September and in the period September-December

4. The proportion of patients who have a medical contact (either scheduled or unscheduled) in September and in the period September-December

5. The total number of medical contacts (either scheduled and unscheduled) per patient in September and in the period September-December

6. The time to first medical contact in September and in the period September-December

7. The proportion of patients who have an unscheduled medical contact in September and in the period September-December associated with a respiratory diagnosis

8. The number of unscheduled medical contacts per patient in September and in the period September-December associated with a respiratory diagnosis

9. The time to first unscheduled medical contact associated with a respiratory diagnosis in September and in the period September-December

10.  The number of prescriptions per patient in the month of August

11.  The number of prescriptions in the 12 months following the intervention

12.  The proportion of patients who have a scheduled medical contact (for example asthma review) in August

13.  The proportion of patients who have a scheduled medical contact (for example asthma review) in the 12 months following the intervention.

The above analyses will be undertaken on patients aged 5 years - 16 years, since asthma is difficult to diagnose in children below this age [16,17]; patients aged < 5 will be analysed separately to these (see the statistical analysis section for more details).

### Setting and site recruitment

The trial will be conducted within primary care with GPs that are currently part of Clinical Practice Research Datalink (CPRD). Invitation letters will be sent out, via CPRD, to all current active GP CPRD sites within England and Wales. 140 sites are expected to be recruited within 6-month (1st January 2013 to 30th June 2013) based on CPRD’s previous experience of recruiting GPs to research trials [12].

Support will also be accessed from the Primary Care Research Network (PCRN) to advertise the trial. Interested practices will be forwarded further information regarding the practicalities of delivering on the study including sign up to CPRD, if they are not already members, as this is a requirement for participation.

### Randomisation

The randomisation will be stratified by size of practice to ensure that there is an equal sample size – in terms of number of school age asthmatic children – in each arm of the trial. The allocation ratio will be 1:1. The randomisation list will be generated using the Stata statistical software. Randomisation will be done centrally: upon confirming their participation, practices will be randomised to one of the two arms; intervention vs usual care. Randomisation will be undertaken by a statistician within the University of Sheffield’s Clinical Trials Research Unit (CTRU), in line with the randomisation plan.

GPs randomised to intervention will be asked to send the postal intervention to eligible patients; GPs randomised to control will continue with usual care and will not be required to do anything further.

### Site compliance

A member of the study team will be responsible for site set up and maintaining on-going contact with the GP setting. Site set up will be done either via telephone or video conferencing. For those sites that prefer a face-to-face contact the study team will either do the visit (if within a reasonable distance) or liaise with the appropriate local PCRN to do the visit on their behalf.

An electronic GP study information pack will be sent out to all sites with a flow chart to show progress through the trial period, timing of intervention, information required from sites and subsequent NHS service support cost payments.

### Withdrawal

Practices are free to withdraw from the trial at any time. This will be documented on a site withdrawal form. Any data already collected during the course of the trial up to the point of withdrawal will be used in the final analysis. With permission from the site data will be retained from the practice as supplied by CPRD.

### Target population for intervention

The population targeted for the intervention will be school aged asthmatic children (aged between 4 and 16 years) registered with a GP.

### Inclusion criteria

Children aged between 4 and 16 years of age as of 1st September 2013 with a coded diagnosis of asthma who have been prescribed asthma medication in the previous 12 months.

### Exclusion criteria

Children aged 4 and under and over 16 years of age as of 1st September 2013, children who are not considered appropriate for this intervention by their GP, and children who are not receiving asthma medication and have no co-existing neoplastic disease.

The CPRD will identify eligible participants based on pre-agreed diagnostic codes for asthma and the inclusion/exclusion criteria. CPRD will send the list of eligible participants for the GPs to check and confirm. The study team will receive anonymised observation data thus informed consent will not be required.

### Trial intervention

A brief postal intervention (letter) has been designed specifically for the PLEASANT trial, developed with input from the study team, which includes a GP, Psychologist and Consultant Respiratory Paediatrician as well as the trial steering committee. It has also been discussed in detail, and reviewed, at two patient and public events that included school aged children with asthma and their parents.

The letter highlights the potential risks associated with stopping, or running low on, asthma medication during the school summer holidays and the effect this may have on exacerbation of asthma when returning to school. It encourages maintaining, or restarting if stopped, medication and to contact the GP or practice nurse if a prescription is required or clarification is needed on the dose. A copy of the intervention/letter is available upon request and will be published on the PLEASANT website from October 2013.

The intervention will be sent, from the GP, to the parents/carers of children with asthma at the beginning of the summer school holidays (week commencing 29th July 2013).

### Data collection

#### Clinical Practice Research Datalink

The CPRD is a computerised database of anonymised longitudinal medical records from primary care [18]. The CPRD are able to capture all medical contacts, from prescription request through to out of hours contacts, along with the reason for the contact. This therefore negates the need to request this information from the GP, reduces practice burden, and ensures complete data sets.

CPRD will extract data from GP medical records 3 times; baseline (NHS contacts for the previous 12 months) and at 1 month and 12 months post intervention. The study team will not have access to any patient identifiable data and will receive all data fully anonymised.

### Allocation of scheduled versus unscheduled contacts

Every NHS service contact is coded by the GP practice and captured within the practice database. Using these codes we will allocate to either scheduled or unscheduled contact.

A scheduled contact is defined as any contact that is part of the planned care for the patient, for example an asthma review; a medical review; repeat prescription or immunisation. An unscheduled contact will be any contact not part of the patient’s care plan that is either patient initiated or as a result of illness.

To ensure the allocation of scheduled and unscheduled contacts are robust an adjudication panel, consisting of 3 GPs, will independently review the prescription and diagnosis codes allocating to either scheduled or unscheduled contact.

### Statistics

#### Sample size

From previous research in the CPRD practice population 30% of school age asthmatic children had at least one unscheduled medical contact within the month of September [13]. We postulate that the intervention may reduce the number of children who have unscheduled medical contacts from 30% to 25% (i.e. an absolute reduction of 5%). This gives an effect size of 5%. The average practice size in the CPRD is 8,294. We thus anticipate circa 100 school-age asthmatic patients per practice (based on 12% of a practice being school age children and 11% of school-age children having asthma). Hence, to detect a difference of 5% with 90% power and two-sided significance level of 5%, with an intra-class correlation (ICC) of 0.03 to account for clustering we require 70 practices per arm. The sample size of 140 practices would equate to approximately 14,000 school age asthmatic patients.

Ukoumunne et al. [19] give estimates of ICCs for patients with respiratory symptoms in symptoms in General Practice. Based on the work of Ukoumunne et al. an ICC of 0.03 is a conservative estimate. The power of the study for ICCs of 0.01, 0.02, 0.03, 0.04 and 0.05 is respectively 99.4, 96.0, 90.0, 83.1 and 76.2%.

As a further sensitivity analysis we will investigate the effect of practices not sending out the letter as planned. Suppose 10 practices failed to send out the letter, these would still be included in the primary analysis under the intent-to-treat principle. However, the effect that could be observed would be reduced to 4.3%. Under the sample assumptions (ICC = 0.03, etc.) the power for the same sample size is reduced to 79.3%. This is a little under 80% but it does demonstrate that the study is reasonably robust to at least one deviation in the planned design.

#### Data analysis

The study periods are defined in three stages. The primary study period is 1st – 30th September 2013, since this is the period when the intervention is felt likely to impact. The extended study period is 1st September - 31st December 2013, since asthma-related appointments are more frequent in the entire period. The follow-up period is 12 calendar months from 1st September 2013 to 31st August 2014.

The primary analyses will be by intent-to-treat among patients aged 5–16 years as of 1st September 2012. The primary endpoint (the proportion of patients who have an unscheduled medical contact in September) will be analysed by logistic regression in which the covariates will include the individual's age; gender; number of contacts the previous September; the trial arm (intervention or control); and the design/cluster effect of general practice as a random effect.

The same approach will be used for analyses based on the extended period. The proportion of patients who have an unscheduled medical contact, the proportion of patients who have any medical contact; and the proportion of patients who have an unscheduled medical contact associated with respiratory illness. The number of unscheduled medical appointments per patient in the extended period; the total number of medical contacts (scheduled and unscheduled) per patient in the extended period; the number of unscheduled medical contacts per patient associated with a respiratory diagnosis in the extended period; the number of prescriptions per patient in the month of August; and the number of prescriptions per patient in the 12 months following the intervention will be analysed in an analogous approach to the primary endpoint. A random effects negative binomial model will be fitted, including the same covariates as above. Further analyses will address the time to first medical contact (defined as the number of days from the start of school term (2nd September 2013) to the date of first appointment, up to and including December 2013, the time to first medical contact up to and including December 2013, and the time to first unscheduled medical contact associated with a respiratory diagnosis. These analyses will be conducted using a random effects ("shared frailty") regression model including the same covariates as described previously.

Patients aged 4–5 will be analysed separately to those aged 5–16, since the diagnosis of asthma is more controversial in this age group; it is often not practical to measure variable airway obstruction below the age of 5 making diagnosis of asthma difficult [16,17]. The impact of the intervention in patients under 5 will be compared to that seen in the main analysis to assess whether the intervention appears to benefit younger children. Additional exploratory analyses will investigate whether the impact of the intervention is related to age or other characteristics of the patient.

A detailed description of the statistical analysis of efficacy and safety outcomes will be written in the trial Statistical Analysis Plan which will be finalised prior to receiving post-intervention data from CPRD. The trial will be reported using the principles highlighted in the CONSORT statement for reporting cluster RCTs [20].

#### Economic evaluation

An exploratory economic evaluation will be undertaken to compare the incremental cost per quality adjusted life year (QALY) of the intervention versus standard care. The perspective of the analysis will be that of the NHS and Personal Social Services. The time horizon will be one year from the intervention and therefore no discounting will be applied.

Data on the number and type of medical contacts in the intervention and control arms will be collected through the CPRD, and combined with PSSRU unit costs [14] and Department of Health reference costs [15] to assess the cost of medical contacts in each arm. Unscheduled contacts in September will be included to capture the impact of any reduction in asthma exacerbations. Scheduled contacts in the year following intervention will be included to capture any change in health care resource use in response to the letter. These will be reported separately in addition to reporting the overall costs of medical contacts for each arm. Prescription costs will be assessed for the year following intervention by combining data on the number of prescriptions with unit cost data from the British National Formulary (BNF). The costs for prescriptions and medical contacts will be combined to give overall costs in the control arm. In the intervention arm the overall cost will also include the cost of sending out the intervention, which will be based on the national primary care costing template from the UK National Institute for Health Research (NIHR) Primary Care Research Network (PCRN) [21].

An existing systematic review [22] has found evidence showing that asthma exacerbations have a significant impact on quality of life [23]. This review will be updated to identify any more recent publications, and literature evidence will be used to determine the health-related quality of life decrement associated with an asthma exacerbation that results in an unscheduled medical contact. From this the QALY gain of preventing asthma exacerbations with be estimated. We will assume that the intervention has no effect on survival and therefore any QALY gain will be wholly driven by improvements in quality of life achieved by preventing asthma exacerbations.

This evidence will inform a simple decision-analytic model to estimate the mean costs and QALYs for the intervention and control groups. Univariate sensitivity analyses and probabilistic sensitivity analyses will be used to examine the uncertainty in the model, with results displayed using cost-effectiveness planes and cost-effectiveness acceptability curves [24].

#### Safety assessments & reporting procedures

The trial intervention is aiming to optimise usual asthma care and improve adherence to medications already prescribed by the GP, thus reducing potential exacerbation of asthma following return to school in September. Therefore involvement in the trial should not result in any adverse or serious adverse events as a result of participation.

Any asthma complications relating to the health of the child would be picked up by their GP or out of hours service and managed as per usual care. These unscheduled/emergency contacts with NHS services will be picked up as part of the routine outcome data and described within the final trial report. Therefore there are no formal reporting procedures, for adverse events or serious adverse events, in place.

Practices randomised to the intervention will be provided with a short reporting template to inform the study team of any incidents they feel are related to the conduct of the trial.

### Trial governance

Two committees are being established to govern the conduct of the study:

1. Trial Management Group (TMG)

2. Trial Steering Committee (TSC)

All committees are governed by Sheffield CTRU standard operating procedures. The TMG consists of the Principal Investigator, co-investigators and key staff within the CTRU. The role of the TMG is to implement all parts of the trial.

The TSC consists of the Principal Investigator, key staff within the CTRU (as non-voting members), an independent chair and two independent members (including a statistician) and 2 lay members. The roles of the TSC are to provide supervision of the protocol and statistical analysis plan, and to provide advice on and monitor progress of the trial. A Data Monitoring and Ethics Committee (DMEC) will not be required for this trial so the TSC will also consider any issues related to patient safety.

### Monitoring arrangements

Once all research governance approvals are in place the study team will contact each GP site for a study set up meeting. This will be before randomisation to check the site has all the necessary information and staff in place before starting the study. Following the intervention there will be a one-off follow-up to ensure the intervention has been delivered within the time specified. No further contact will be required from the practices.

### Ethical considerations

The study has been approved by the South Yorkshire Research Ethics Committee on 25th October 2012 reference: 12/YH/0478 and will be conducted in accordance with the Research Governance Framework for Health and Social Care 2005 [25].

### Patient and public involvement

The PLEASANT trial research team is committed to the principles of patient and public involvement (PPI) [26] and have ensured that best practice on PPI has been followed [27]. Children with asthma and their parents have been involved in the design of the trial and will be involved throughout the conduct of the study.

### PPI in the design of the trial

During the design of the trial, a PPI consultation event was held in January 2011 with a group of children with asthma and their parents. This event was used to investigate whether the hypothesis underlying the trial was supported by children with asthma and their parents, to give an opportunity for the children and parents to discuss the wording of the intervention (the GP letter) and to give their views on to whom the letter should be addressed (i.e. should it be addressed to the child or to their parent/guardian). This initial PPI consultation event was written up as a University of Sheffield report [28].

### PPI throughout the trial

PPI during the conduct of the study will have two components: (1) two PPI consultation events, the first held in September 2012 (written up as a University of Sheffield report [29]) and one planned for December 2014/January 2015, involving up to 6 children and their parents/guardians; (2) parents of children with asthma will be invited to become members of the TSC (of which three accepted), which will meet twice in the first year and once in the final year of the study.

For attending the consultation events, each child will be provided with a £20 gift voucher and their parents will be able to claim for their expenses such as travel.

Parents of children with asthma on the TSC will be paid for their time at a rate of £50 per meeting, and will receive travel expenses. The study’s PPI lead will offer to meet with the parent members of the TSC before or after each meeting to discuss the agenda items and any issues of concern, and will act as a mentor for them.

### Finance and indemnity

The trial has been financed by the NIHR Health Technology Assessment (HTA) Programme. This is an NHS sponsored study. If there is negligent harm during the clinical trial when the NHS body owes a duty of care to the person harmed, NHS indemnity will cover NHS staff and those conducting the trial. The trial will be conducted in accordance with the Medicines for Human Use (Clinical Trials) Regulations (SI2004/1031) [30].

The University of Sheffield has in place insurance against liabilities for which it may be legally liable and this cover includes any such liabilities arising out of this clinical trial.

### Independent scientific review

The trial has been independently reviewed by the HTA prior to funding and by the Independent Scientific Advisory Committee (ISAC) for Medicines and Healthcare products Regulatory Agency (MHRA) database research.

### Reporting and dissemination

Results of the trial will be disseminated in peer reviewed scientific journals and clinical and academic conferences. A patient and public event will also be held to feedback to those involved in the development of the study protocol.

Details of the trial will also be made available on the study website [31]. Summaries of the research will be updated periodically to inform readers of the on-going progress.

## Discussion

The PLEASANT trial will assess whether a brief postal intervention from the GP can reduce unscheduled appointments and exacerbation of asthma in school aged children associated with the return to school after the summer holidays. It has the potential to benefit the health and quality of life of children with asthma while also improving the effectiveness of NHS services by reducing NHS use in one of the busiest months of the year.

An exploratory health economic analysis will gauge any cost saving associated with the intervention and subsequent impacts on quality of life. If results for the intervention are positive it is hoped that this is could be adopted as part of routine care management of childhood asthma in general practice.

GP recruitment began in January 2013 with the intervention due to go out at the beginning of the school summer holidays in July 2013. Observational data will be collected from approximately 14000 children with final data collection in December 2014. The final study report will be written up by June 2015.

## Trial status

The trial is currently open and undergoing GP site recruitment.

## Abbreviations

BNF: British national formulary; CI: Confidence interval; CTRU: Clinical trials research unit; CPRD: Clinical practice research datalink; DMEC: Data monitoring and ethics committee; GP/s: General practice/s; ICC: Intra class correlation; ISAC: Independent scientific advisory committee; MHRA: Medicines and healthcare products regulatory agency; NHS: National health service; NIHR: National institute for health research; PLEASANT: Preventing and lessening exacerbations of asthma in school-age children associated with a new term; QALY: Quality adjusted life year; PCRN: Primary care research network; PPI: Patient and public involvement; PSSRU: Personal social services research unit; TMG: Trial management group; TSC: Trial steering committee.

## Competing interests

The authors declare they have no competing interests.

## Authors’ contributions

MJH and SAJ are the Trial Manager/co-investigator and Chief Investigator of the trial respectively. SAJ conceived the study. MJH, SAJ, CLC, HE, HWS, TvS participated in the design of the trial. JB is lead for PPI aspects of the study. MJH, SAJ, HE, PN, WHS were involved in the development of the intervention. MJB is responsible for the statistical analysis and SD the health economics. TvS will oversee site recruitment and data collection for CPRD. All authors were involved in the funding application, development of the trial protocol and are members of the Trial Management Group. MJH drafted the manuscript all other authors have provided review. All authors have read and approved the final manuscript.
